# Free vascularized fibula transfer in single- or double-barrel technique for reconstruction of segmental bone defects following oncological resection or posttraumatic bone loss

**DOI:** 10.3205/iprs000189

**Published:** 2024-12-09

**Authors:** Kevin Bienger, Vlad Stefan, Adrian Dragu, Olimpiu Bota, Feras Taqatqeh, Klaus-Dieter Schaser, Michele Rudari, Hagen Fritzsche

**Affiliations:** 1 University Center for Orthopedics, Trauma and Plastic Surgery, Department of Plastic and Hand Surgery, University Hospital Carl Gustav Carus at the TU Dresden, Germany; 2 Department of Plastic Surgery, First Surgical Clinic, Emergency County Hospital Cluj-Napoca, Iuliu Haţieganu University of Medicine and Pharmacy, Cluj‑Napoca, Romania

**Keywords:** free vascularized fibular bone graft, bone reconstruction, bone tumors, segmental bone loss, segmental bone defect, orthopaedic surgery

## Abstract

**Background:**

Significant osseous defects or osteonecrosis, precipitated by open fractures, infections, or neoplastic conditions, represent infrequent yet critical medical conditions. The free vascularized fibular graft (FVFG) is a challenging but straightforward, reliable surgical intervention for the reconstruction of defects across various anatomical regions. This study aims to compare, quantify, and demonstrate the FVFG’s versatility. The utilization of a single- or double-barrel approach, contingent on the defect’s characteristics, optimizes length conservation or enhances the stability of extensive defects.

**Methods:**

We retrospectively evaluated patients who underwent the FVFG procedure, employing either a single- or double-barrel technique, at our medical center during the period from August 2017 to May 2023. The inclusion criterion was the presence of substantial osseous defects (bone loss in straight bone over 8–10 cm or multi-level spine resection), precipitated by trauma, neoplasms, non-union fractures or infections.

**Results:**

Our study encompassed eight male patients, with an average age of 31 years, ranging from 10 to 56. Each patient underwent osseous resection due to osteomyelitis (n=2), tumor excision (n=4), or pseudarthrosis (n=2) resulting from previous trauma, followed by a free fibula flap as part of the FVFG procedure. When fibula was simultaneously prepared already during tumor resection (n=2), there was a significant reduction in the overall operation time. Postoperative assessment revealed that full osseous integrity without any graft failure was restored in all patients, and the same number of patients regained independent ambulatory ability. Surgical complications were observed in one patient, who exhibited wound healing post-reconstruction, all of which were rectified through subsequent surgical intervention.

**Conclusion:**

Diverse osseous defects in complex cases can be reconstructed by using the FVFG, thereby restoring maximal reconstructive capacity, expedited union compared to non-vascularized bone, and acceptable donor site morbidity. FVFG remain a reliable solution for diverse defects. Moreover, in cases requiring complex tumor defects, careful preoperative planning and an interdisciplinary treatment are essential for successful treatment.

## Zusammenfassung

**Hintergrund:**

Knochendefekte oder Osteonekrosen, die durch offene Frakturen, Infektionen oder neoplastische Erkrankungen hervorgerufen werden, sind seltene, aber kritische medizinische Zustände. Das freie vaskularisierte Fibulatransplantat (Free Vascularized Fibular Graft, FVFG) ist ein anspruchsvoller, aber zuverlässiger chirurgischer Eingriff zur Rekonstruktion von Defekten in den verschiedensten Regionen des menschlichen Skeletts. Ziel dieser Studie ist es, die Vielseitigkeit des FVFG zu vergleichen, zu quantifizieren und zu demonstrieren. Die Verwendung eines Single- oder Double-Barrel FVFG, abhängig von den Defektcharakteristika, optimiert den Längenerhalt oder verbessert die Stabilität von ausgedehnten knöchernen Defekten.

**Methoden:**

Wir haben retrospektiv die Patienten ausgewertet, die im Zeitraum von August 2017 bis Mai 2023 in unserem medizinischen Zentrum mit einer Single- oder Double-Barrel-Technik am FVFG operiert wurden. Das Einschlusskriterium war das Vorhandensein eines knöchernen Defekts, ausgelöst durch Traumata, Neoplasien, Nonunion-Frakturen oder Infektionen.

**Ergebnisse:**

Unsere Studie umfasste acht männliche Patienten mit einem Durchschnittsalter von 31 Jahren und einer Altersspanne von 10 bis 56 Jahren. Jeder Patient unterzog sich einer knöchernen Resektion aufgrund einer Osteomyelitis (n=2), einer Tumorexzision (n=4) oder einer Pseudarthrose (n=2) infolge eines früheren Traumas, gefolgt von FVFG. Wurde die Fibula bereits bei der Tumorresektion (n=2) präkonditioniert, so verkürzte sich die Gesamtoperationszeit signifikant. Die postoperative Beurteilung ergab, dass bei allen Patienten die vollständige knöcherne Integrität ohne Versagen des Transplantats wiederhergestellt wurde, und ebenso erlangten alle Patienten ihre unabhängige Gehfähigkeit zurück. Chirurgische Komplikationen wurden bei einem Patienten beobachtet, bei dem es nach der Rekonstruktion zu einer Wundheilung kam, die jedoch durch einen weiteren Eingriff behoben werden konnte.

**Schlussfolgerung:**

Knochendefekte in komplexen Fällen können mit FVFG rekonstruiert werden, wodurch eine maximale Rekonstruktionskapazität, eine schnellere Heilung im Vergleich zu nicht vaskularisierten Knochen und eine akzeptable Hebemorbidität erreicht werden. FVFG sind nach wie vor eine zuverlässige Lösung für verschiedene Defekte. Darüber hinaus sind bei komplexen Tumordefekten eine sorgfältige präoperative Planung und eine interdisziplinäre Behandlung für eine erfolgreiche Behandlung unerlässlich.

## Introduction

The task of reconstructing substantial osseous defects, precipitated by trauma, neoplasms, non-union fractures or infections, presents a formidable clinical challenge. The selection of the appropriate surgical technique is contingent on the defect’s etiology, dimensions, and biomechanical requirements. A decennial fracture registry at a level-1 trauma center revealed that around 0.4% of all fractures involved significant bone loss [1], [2], [3], [4], [5]. Patients treated with autologous bone grafts (such as iliac crest grafts) or allogeneic spongiosa, supplemented with osteosynthesis, exhibited favorable outcomes. However, these methodologies are deemed unsuitable for cases with large, segmental defects or individual complicated scenarios, subsequent to infection or irradiation [1], [2], [3], [6].

The free vascularized fibular graft (FVFG) presents a viable solution for bridging substantial osseous defects, facilitating the administration of elevated concentrations of systemic antibiotics, and restoring an appropriate geometric profile [7], [8], [9], [10]. Numerous studies have documented the successful reconstruction of extensive defects due to prior infection, and the bridging of significant osseous defects in both the upper and lower extremities [5], [7], [8], [11]. The overall incidence of critical-sized bone defects – for example in straight bone up to 10–15 cm [12] – remains low and usually necessitates more intricate reconstruction techniques [4], [5], [6].

The fibula, a long, straight bone (up to 30 cm in length) with a three-cortical structure, can be conveniently inserted into the intramedullary canal of larger bones. It can be harvested as a chimeric osteo-fasciocutaneous flap or solitary bony graft. Donor-site morbidity is minimal, given that the fibula bears only a minor load over the ankle, particularly if efforts are made to preserve the distal 6 cm of the fibula during harvest to avert ligamentous/syndesmotic ankle instability or deformity [5], [13].

The fibula benefits from both endosteal and periosteal blood supply, predominantly from the peroneal artery. The peroneal artery, a branch of the posterior tibial artery, originates approximately 2–3 cm distal to the popliteus muscle and courses along the medial edge of the fibula, nestled between the flexor hallucis longus and tibialis posterior muscles. The endosteal blood flow is derived from a nutrient vessel that penetrates the bone in the posteromedial aspect of the middle third of the fibula, at an average distance of 17 cm (range, 14–19 cm) below the styloid process, and is deemed the primary blood supply [6], [14]. The ensuing blood flow is centrifugal, coursing from the medulla to the cortex. The periosteal blood flow is copious and reticulated, nourishing the outer third of the cortex, and is also supplied by the peroneal artery. This abundant periosteal blood supply allows multiple osteotomies, as evidenced in head and neck reconstruction [6], [14], [15]. It is deemed the secondary blood supply. The peroneal pedicle, with a length of 6–8 cm and an arterial diameter of 1.5–3 mm, is flanked by accompanying veins. As a general rule, recipient vessels should be situated outside the injury zone and ensure forward flow to the flap [6], [14].

This study aims to compare, quantify, and demonstrate the FVFG’s versatility. The utilization of a single- or double-barrel approach, contingent on the defect’s characteristics, optimizes length conservation or enhances the stability of substantial bone loss subsequent to bone resection due to tumor, non-union fracture healing, or osteomyelitis. We also propose a strategy for managing instability and administering elevated concentrations of systemic antibiotics in this setting.

**Figure 1:**
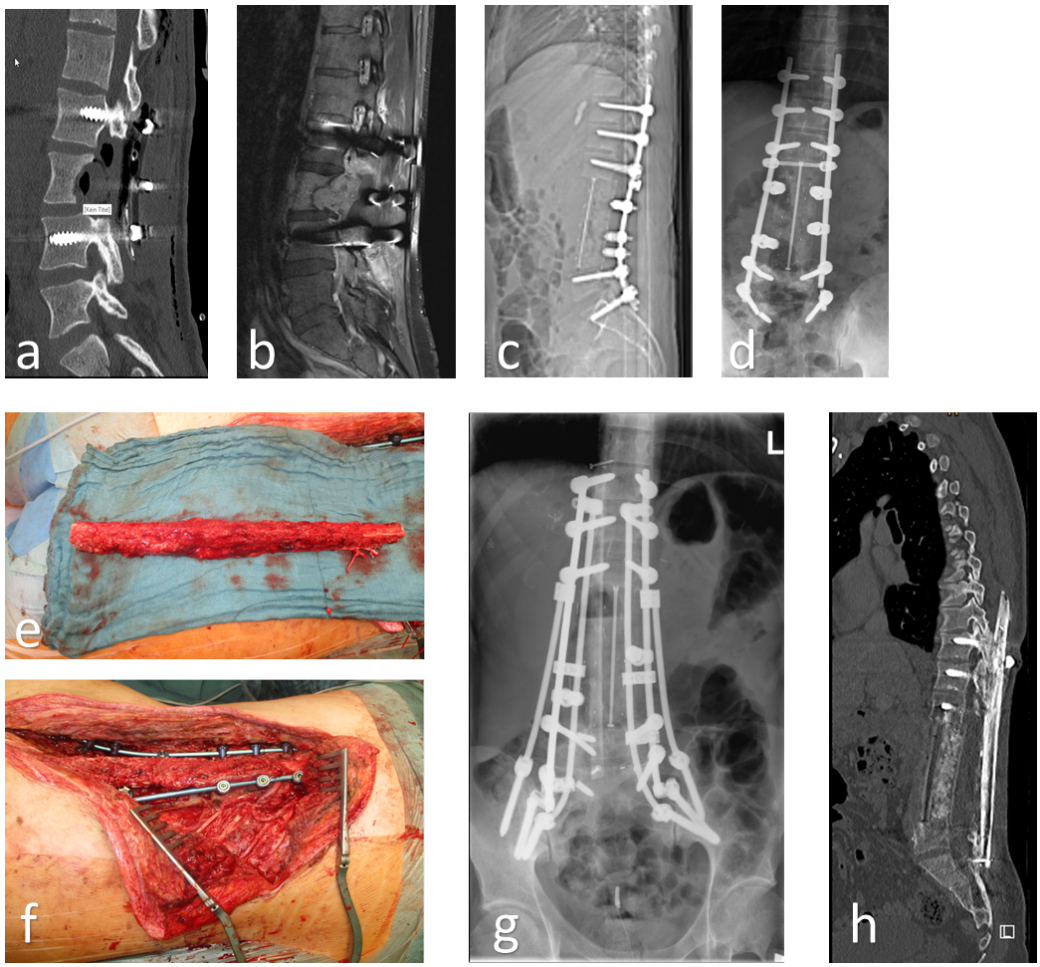
26-year-old male with high-grade osteosarcoma and 3-level en bloc resection of the lumbar spine after external decompression for paraplegia and neoadjuvant chemotherapy (a–c). 2 years later, no evidence of recurrence or metastases. However, mechanical failure of the instrumentation (d). Implant removal, spino-pelvic/ilio-lumbar/thoraco-lumbar re-instrumentation from Th11/12/L1 to L5/S1 as well as bilateral ilium with triple rod construction, TLIF L5/S1, dorsal, bony spondylodesis using vascularized autologous fibula transfer (graft length 26 cm) from Th11 to sacrum, fixation of the graft using small fragment screw osteosynthesis thoracically to the processus spinosus LWK 2 lumbar/sacral to the lamina S1 (e/f). 2 years postoperatively consolidated fibula, stable instrumentation, no complaints, no recurrence or metastases (g/h).

A single-barrel FVFG can be employed in any circumstance necessitating the reconstruction of large skeletal defects with proximal and distal fixation, with the potential for additional osteosynthesis. A double-barrel FVFG can be used to achieve a higher degree of stability, such as in the case of an en-bloc resection of parts of the spine (Figure 1a/b (Fig. 1)).

FVFG can be successfully employed in the reconstruction of extremity long bones (femur, tibia, humerus, radius or ulna) as well as in the context of extensive spinal and pelvic defects. Certain contraindications for the use of FVFG include incomplete tumor excision, preoperative Doppler studies or angiography revealing significant atherosclerotic disease, or abnormal lower extremity vasculature. FVFG can be applied in infected regions (such as those with a history of osteomyelitis) due to its ability to provide a blood supply with an effective antibiotic concentration, and it also offers substantial length allowing to bridge between healthy areas [7], [11].

Alternative sources of vascularized osseous flaps for smaller defects include free iliac crest, ribs and scapula flaps. However, these do not permit the spanning of large defects, which is possible when using a FVFG. Their success hinges on the availability of a suitable vascular access point, which may not always be present.

## Material and methods

In this retrospective cohort study, all patients who underwent microsurgical intervention between August 2017 and May 2023 for the treatment of substantial bone defects or losses caused by open fractures, infections, or neoplastic diseases were included (bone loss in straight bone over 8–10 cm or multi-level spine resection).

The clinical investigation was conducted in accordance with the Declaration of Helsinki and underwent review by the Ethics Committee of the Technical University of Dresden (EC 219052023). All data collection and processing procedures were anonymized.

The study comprised eight male patients, with an average age of 31 years (ranging from 17 to 56 years), who exhibited a substantial bone defect following a tumor free margins resection, osteomyelitis, or fracture with atrophic non-unions.

For all patients, wound swabs were collected for bacterial culture and antibiograms, inflammation blood tests (including C-reactive protein, erythrocyte sedimentation rate, and procalcitonin) were conducted, and a histopathological examination was performed to determine the tumor stage. Microbiological confirmation of bone samples (at least 3–5 samples) and additional histopathological processing confirmed the presence of osteomyelitis.

### Surgical procedure

Prior to the flap harvest, both the donor and recipient sites warrant meticulous evaluation. A comprehensive medical history should be obtained, specifically inquiring about lower limb issues such as intermittent claudication, deep vein thrombosis, lower limb trauma, and varicose veins. The strength of both the dorsalis pedis and posterior tibial pulses should be assessed, along with the condition of the lower limb skin. In instances of abnormal pedal pulses or significant lower limb trauma, a computed tomography angiography of the donor leg should be performed preoperatively to rule out the presence of peroneal artery occlusion, ideally three vessel perfusions. The residual soft tissue coverage at the donor site should be evaluated, and a chimeric osteocutaneus flap should be harvested if necessary. The recipient vessels should undergo clinical examination and Doppler ultrasound, supplemented with a digital subtraction angiography if concerns arise. The defect’s size and location (considering potential further removal of diseased tissue) will dictate the required size and shape of the flap. In tumor resection cases, an external or occasionally a temporary internal fixator is installed prior to any removal to accurately restore bone length and width later (after spinal en bloc excisions an internal fixator/screw-rod construct is inevitably needed throughout the entire reconstruction period and thereafter). To facilitate access during surgery, the frame can be removed with the pins left intact (not in patients after en bloc vertebrectomies); reattaching the fixator post-removal will enable the restoration of anatomical parameters. The surgical technique is partitioned into three distinct phases: recipient site preparation, graft harvest, and defect reconstruction [6], [14].

**Figure 2:**
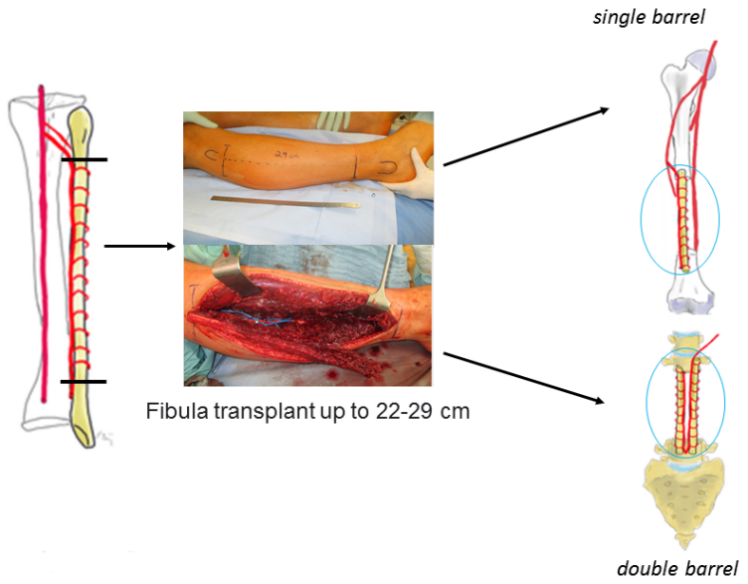
Schematic process of harvesting the FVFG as a single- or double-barrel

Once the vascular pedicle is exposed, the lower limb tour-niquet is released with the graft in situ until the recipient site is prepared, to minimize ischemia time. The incision over the recipient site is strategically planned to circumvent areas of compromised skin and ease bone exposure and dissection of the recipient vessels [14]. The pathological defect is progressively excised until the margins of healthy bone are attained. The recipient vessels are scrutinized for any defects, which could potentially impede blood flow. In such instances, the anastomosis should be performed more proximally and may necessitate vein grafts if the pedicle length proves insufficient. Once the recipient site is prepared, the fibula is harvested by ligating the peroneal vessels and detaching its residual posteromedial connection to the flexor hallucis longus. Once detached, the osteotomy is performed and the fibula is folded through the middle resulting in double-barrel structure (Figure 2 (Fig. 2)). A single-barrel structure doesn’t necessitate the osteotomy and folding of the fibula.

The creation of a double-barrel structure involves performing closing wedge osteotomies at the midpoint along the length of the graft. Folding of the fibula is performed on the same side as the pedicle to prevent tension on the vascular supply. During the osteotomy process, it is crucial to carefully separate and safeguard the periosteum and vessels from the fibula. It is important to strategize such that the pedicle is not strained following the osteotomy and positioning of the fibula. The bony ends are aligned, and each extremity is secured either with a screw/plate osteosynthesis or in spinal/pelvic localisations using internal fixator/screw rod constructs. Alternatively, the FVFG is then inserted into the bone defect, with fixation performed at both the proximal and distal ends [6], [14]. The best procedure is to drill holes with a drill or K-wires into the free bone ends of the original bone to address petechial bleeding, and then bolt the fibula, either press-fit anchored or additionally with one screw proximally and distally, each tetracortical. The procedure concludes with the closure of the wound.

All patients adhered to the same postoperative protocol. Subcutaneous administration of enoxaparin sodium twice daily (4,000 U. I.) was continued until full weight-bearing capacity was regained on the the recipient site. The sutures were removed on the 14^th^ postoperative day. At the donor site, unrestricted weight bearing was allowed from the second day following surgery. 

The technique’s clinical and radiological outcomes were evaluated. The radiological assessment was conducted by our institution’s radiologist. Regular radiological examinations were an essential part of the clinical follow-up. The criteria for assessing patient satisfaction included the absence of infection signs, alleviation of pain, restoration of daily life activities, the ability to walk independently, and overall patient satisfaction.

The results of the data were analysed with Excel 365 (Microsoft Corporation, WA, USA) and SPSS (SPSS Inc., PASW Statistics for Windows, Chicago, USA). The comparison of the data was mainly descriptive due to the small number of cases.

## Results

The study incorporated eight patients, all of whom were male. The patients’ average age was 31 years, with a range of 17 to 56 years (Table 1 (Tab. 1)). Five patients had undergone tumor surgery, while the rest had secondary osteomyelitis defect reconstruction due open fracture (Table 1 (Tab. 1)).

**Figure 3:**
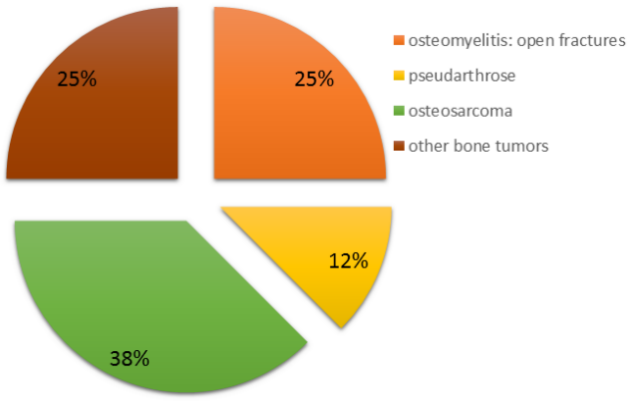
Indication for reconstruction using an FVFG

Within our patient cohort, five patients exhibited bone defects attributable to oncological resections, with osteosarcoma accounting for 60% of the underlying tumor diseases. Tissue samples procured immediately post-resection tested positive for osteomyelitis in all patients with an initial traumatic genesis (Figure 3 (Fig. 3)).

The pain level in the visual analogue scale (VAS) was low both preoperatively and postoperatively (median 3, range 1–3, Table 2 (Tab. 2)).

Complete clinical and radiological bone healing was achieved in 7 out of 8 cases (87.5%), with an average radiological consolidation duration of 9 months (range 7–12 months; Table 2 (Tab. 2)). An equal number of patients were treated with a single- or double-barrel FVFG (Table 1 (Tab. 1)). The receiver site was in five times the lower extremity, twice the spine and in a single case the pelvis.

In two cases the fibula was simultaneously prepared already during tumor resection (two seperated teams using completely different instruments), but not harvested. This procedure resulted in a reduction of the overall operation time of 198 ± 20 min compared to the other six cases and the burden on the patient.

Using a hand-held Doppler probe, no arterial or venous thrombosis of the arterial pedicle were registered.

**Figure 4:**
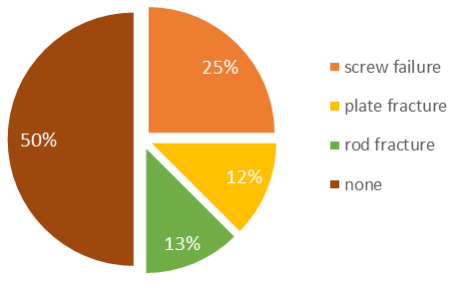
Implant failure cause: revision surgery was required in 50% of patients. However, a complete failure of the FVFG could not be confirmed.

The sole case of failure was a nonunion of the femur, which was rectified by resecting the formed pseudarthrosis and the insertion of additional spongiosa with bone morphogenetic proteins (BMP). Implant failure necessitating revision surgery was encountered in 50% of the patients. However, complete failure of the FVFG could not be confirmed (Figure 4 (Fig. 4)). All four patients underwent a reoperation for the replacement of screws, plates, or rods, even though the graft was fully consolidated. One patient experienced non-union at the distal femur with distal screw failure. After revision with implantation of additional autologous cancellous, full integration and osseous consolidation were visible in the 1-year follow-up. In all instances where bone healing was achieved, patients were able to attain a satisfactory quality of life and resume their work and sporting activities.

Radiological follow-up of the donor site revealed no differences between the harvesting of the dominant leg or the contralateral side. The imaging follow-up examinations in patients demonstrated normal stabilization of the lower limb of the donor site. 

At the donor site, five times of the dominant leg and three times of the non-dominant leg, the duration to return to normal walking activity was around 128 days (Table 2 (Tab. 2)).

## Discussion

Substantial bone defects, caused by post-traumatic osteomyelitis, trauma, tumors, or non-union fractures, necessitate an assertive surgical strategy for successful management [5], [14], [16]. The principle of dead-space obliteration and neovascularization of the affected area has been deemed essential for successful disease management [1], [2], [3]. Following extensive excision of such bone pathologies, reconstructive alternatives for large bone defect surgery encompass the utilization of non-vascularized bone autografts, arthrodesis, allografts, custom endoprostheses, distraction osteogenesis using Ilizarov’s procedure as well as vascularized bone grafts [1], [2], [3], [6], [16], [17].

Literature reports indicate that non-autologous and non-vascularized techniques, when applied in highly infected areas, exhibit high failure rates [3], [18], [16]. These failures can lead to non-union, resorption, restricted joint movements, instability, and inconsistent long-term durability. Conversely, microvascular free tissue transfer has proven effective in providing the requisite tissue bulk and neovascularization for reconstructing the defect resulting from radical debridement [5], [6], [9], [16], [17].

As previously mentioned, there are multiple techniques available for reconstructing large bone defects following bone resection. Literature reports suggest that FVFG hold several advantages over non-vascularized reconstruction techniques. These advantages encompass tolerance to therapeutic levels of postoperative radiation therapy, rapid consolidation, resistance to infection, and hypertrophic reaction [6], [19], [20]. The objective of this study was to ascertain the postoperative outcomes and complication rates in patients who underwent resection for a large bone defect and subsequently encountered issues of stability or significant cortical loss. Consequently, the reconstruction of these defects was undertaken using the double- or single-barrel technique FVFG.

In the context of large defects, when the conventional use of non-vascularized bone in conjunction with osteosynthesis, or treatment with an Ilizarov Fixator, for instance, at the tibia shaft, fails to yield the anticipated results, we opted for the large single-barrel FVFG. Other studies have indicated that the treatment with an Ilizarov Fixator is only suitable for a selected group of patients and does not offer a short term and timely solution. The treatment in average spans several months and is not appropriate for every patient [21], [22].

**Figure 5:**
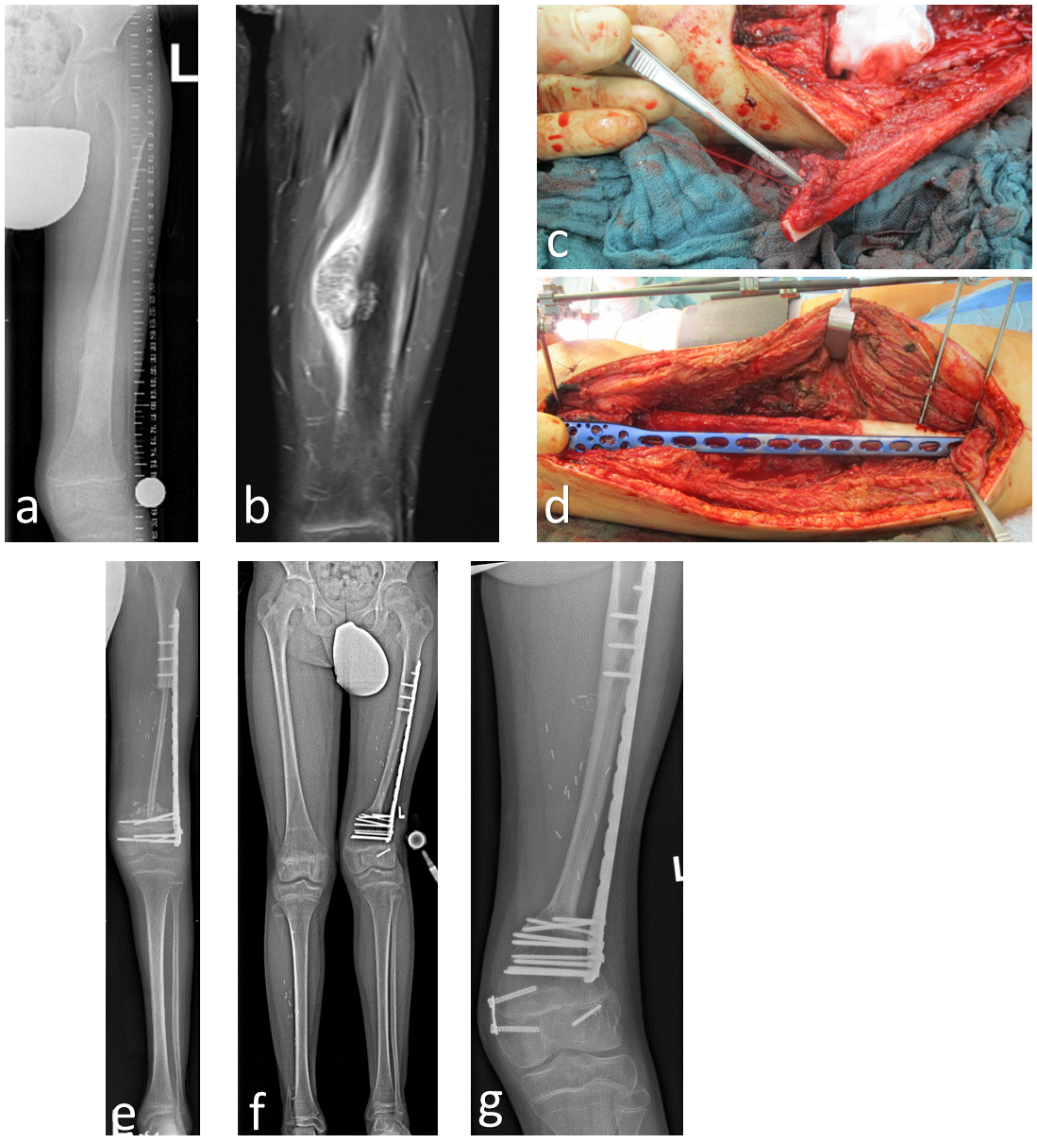
10-year-old male with high-grade osteosarcoma of the femoral shaft on the left side (a/b). After neoadjuvant chemotherapy, en bloc tumor resection and reconstruction with a single free vascular pedicled fibula (from the right side) using microsurgical techniques, cancellous bone grafting and angle-stable plate osteosynthesis (Philos, Depuy Synthes) (c/d). Initial single FVFG (e), 3 years postoperatively, significant hypertrophy with good consolidation (f). After temporary release of the growth plate in the course of genu valgum on the left, an 8-plate was implanted in the left knee joint to gradually guide growth in the case of progressive genu valgum (g). The leg length discrepancy on the left is to be corrected using a magnetically driven intramedullary nail after completion of longitudinal growth.

Owing to the fibula’s capacity to provide more than 30 cm of straight (can be harvested en bloc with the fibula head), healthy bone with minimal morbidity at the donor site and minimal postoperative functional limitation, the use of FVFG demonstrates significant potential for bridging various segmental defects of long bones [23]. This was corroborated in our study in the context of the tibia and femur. Subsequently, accelerated osteointegration was observed, and partial weight-bearing (contact with the sole of the foot) was promptly achieved (Figure 5 (Fig. 5)).

In terms of stability, we observed a failure when using non-vascularized bone (for example Iliac crest bone graft and smaller general hospitals) in conjunction with osteosynthesis. In two patients, the osteosynthesis refractured. Consequently, we opted for the double-barrel technique in patients with adequate fibula length. Literature reports suggest that the double-barrel technique can enhance the weight-bearing load of the reconstruction as well as a better limb function, better postoperative mental and psychological status, and lower complication incidence [24]. The results of this study, which demonstrated early achievement of partial or full weight-bearing, align with those of other studies [8], [25]. The double-barrel technique proves successful for repairing femoral defects, which pose a particular challenge due to the greater cross-sectional mismatch of the femur [6]. However, we noted that patients, particularly those who underwent tumor resections at the spine or femur levels, were more susceptible to osteosyntheses failure than those with posttraumatic resections, with no difference in final union rates. To bolster stability and support, FVFG have recently been augmented with special posterior spinal stabilization or an additional double plate osteosynthesis. Nonetheless, the vascular component of the FVFG remains consistent with that described in other reconstructions [26], [27].

The harvesting, placement, and vascular anastomosis of an FVFG procedure are known to extend operative time by approximately 3–4 hours [19]. Therefore, in selected patients, the fibula was prepared already during tumor resection, but not harvested. This implies that the tumor resection was performed by the tumor surgeons in an interdisciplinary setting, and parallel to this, the fibula was prepared without separation of the pedicle. In the second operation in case of tumor free margins, FVFG transplantation and osteosynthesis were performed. According to literature reports, harvesting of FVFG under tourniquet of the thigh results in negligible additional blood loss from this procedure [9].

Late donor-site morbidity, such as chronic pain, gait abnormality, ankle instability, and sensory deficit, has been described in the literature [28]. In this study, no patients had complications at the donor site of the FVFG, which could potentially diminish the quality of results.

This work has some limitations. The limited use of FVFG, a direct head-to-head comparative study between FVFG and non-vascularized bone grafts, i.e. the relatively low patient number and the retrospective nature of the study resulted in limited data being available for collection, and in some cases, variables were missing. However, all primary outcome variables were present, and no patient was lost to follow-up. This study was performed at only one single-center and may not reflect the experience of other hospitals. Finally, multivariate analysis could not be performed due to the small number of patients included in this study.

## Conclusion

The Free Vascularized Fibular Graft (FVFG) plays a crucial role in the reconstruction of various bony defects. Consequently, the FVFG emerges as a viable and in some cases better alternative for the reconstruction of bony defects compared to non-vascularized bone, given its superior reconstruction capacity, rapid union, and acceptable of donor site morbidity. The employment of double-barrel techniques offers a stable alternative for large defects, particularly in the load-bearing zones of large tubular bones.

However, additional studies are necessary to fully evaluate the efficacy of this technique and to demonstrate a direct head-to-head comparative between FVFG and non-vascularized bone grafts.

## Notes

### Institutional review board

The study was conducted in accordance with the Declaration of Helsinki and was approved by the Ethics Committee of the University of Dresden (EC 219052023).

### Informed consent

Written informed consent for publication has been obtained from the patients.

### Data availability

The datasets analyzed in this manuscript are not publicly available. Requests to access the datasets should be directed to the corresponding author.

### Acknowledgments

We acknowledge support by the Open Access Publication Fund of the University of Dresden.

### Competing interests

The authors declare that they have no competing interests.

## Figures and Tables

**Table 1:**
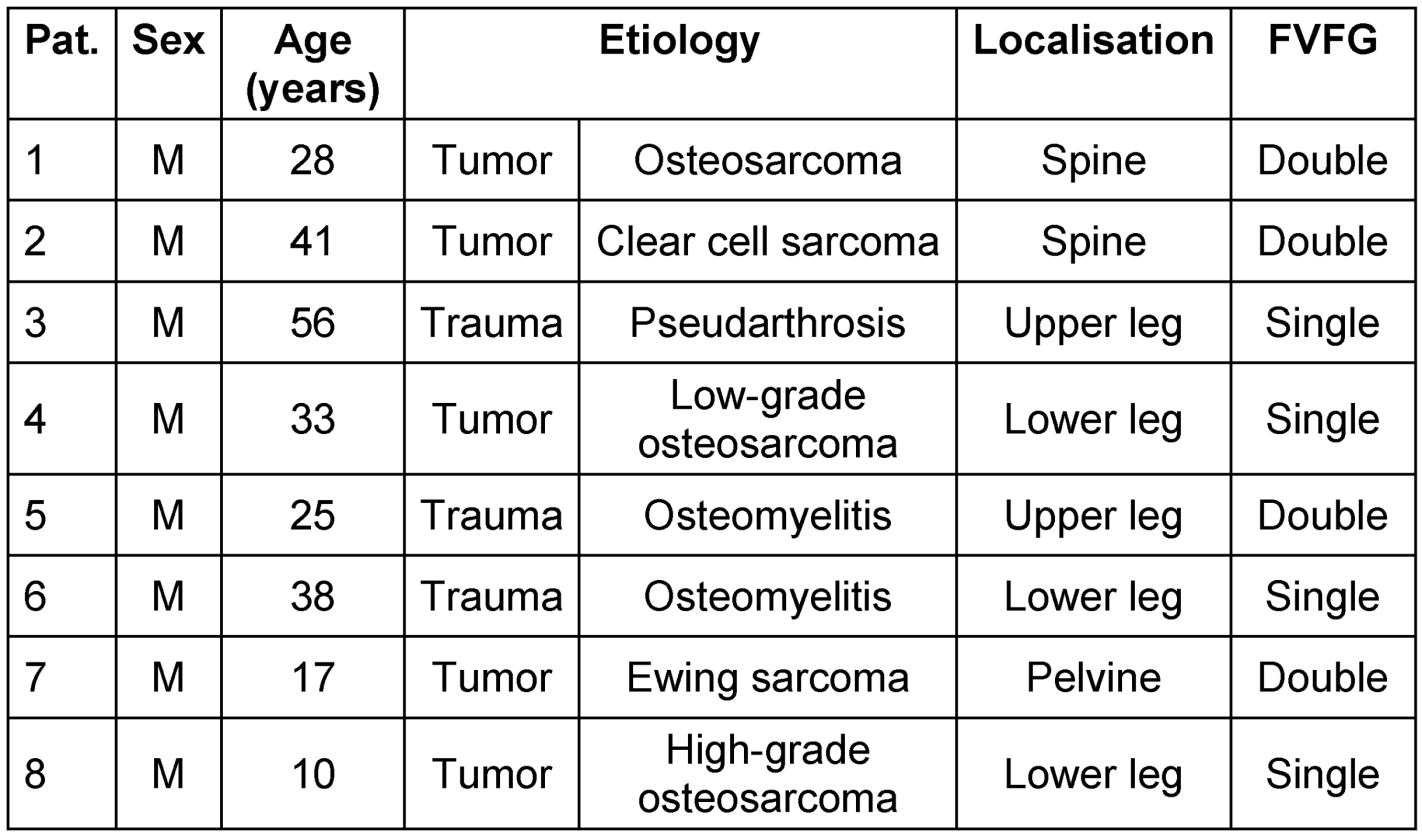
Descriptive data, etiology and location of the bony defect and type of reconstruction

**Table 2:**
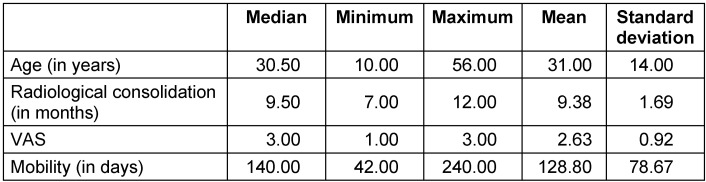
Duration until full weight bearing and radiological consolidation
